# Non-use of contraception: determinants among Ugandan university students

**DOI:** 10.3402/gha.v5i0.18599

**Published:** 2012-10-08

**Authors:** Devika Mehra, Anette Agardh, Karen Odberg Petterson, Per-Olof Östergren

**Affiliations:** 1 Social Medicine and Global Health, Department of Clinical Sciences, Lund University, Malmö, Sweden; 2 Centre for Adolescent Health, Royal Children's Hospital, Murdoch Children's Research Institute, Department of Paediatrics, University of Melbourne, Victoria, Australia

**Keywords:** contraception, Uganda, sexual debut, gender, pregnancy, sexual behaviour

## Abstract

**Background:**

In Uganda, adolescent pregnancy often results in adverse maternal and neonatal health outcomes. In this context, low use of contraception and high rates of maternal mortality rate make preventing unwanted pregnancies critical.

**Objective:**

The objective was to determine the relationship between non-use of contraception and socio-demographic factors, alcohol consumption, and types of partner(s) among Ugandan university students.

**Design:**

In 2010, 1,954 students at Mbarara University of Science and Technology in southwestern Uganda participated in a cross-sectional study whereby a self-administered questionnaire was used to assess socio-demographic factors, alcohol consumption, and sexual behaviour including the use of contraceptives. Multivariable logistic regression was used for the analysis and data were stratified by sex.

**Results:**

1,179 students (60.3% of the study population) reported that they were sexually active. Of these, 199 (18.6%) did not use contraception in their last sexual encounter. Students currently not in a relationship had higher odds of non-use of contraception (odds ratio 1.8, 95% confidence interval 1.2–2.7). The association remained statistically significant for both males and females after controlling for age, sexual debut, area of growing up, and educational level of the household head. Socio-demographic determinants of age (22 or younger), early sexual debut (at age 16 years or earlier), and a rural background were significant for males but not for females. A synergistic effect between not currently being in a relationship and early sexual debut were also observed to have an effect on the non-use of contraception.

**Conclusion:**

Non-use of contraception among Ugandan university students differs for males and females, possibly due to gendered power relations. Sexual and reproductive health policies and programmes should be designed to take these differences into account.

More than half of the world's population is less than 25 years old and approximately 85% of this demographic segment lives in low- or middle-income countries (1). The sexual behaviour of such young people has become a crucial social and public health concern, especially with regard to unintended pregnancies. It is estimated that 41% of all pregnancies globally are unintended and 39% occur in Africa (2). According to World Health Organization (WHO), the lifetime risk of death due to pregnancy is 1:22 in sub-Saharan Africa, with adolescents facing a higher risk of morbidity and mortality than older women (3).

The bio-social gap, explained as the early onset of puberty and increasing age of marriage, has widened in most low-income countries (4). This has led to an increase in pre-marital sexual activity, which exposes vulnerable youth to the risk of unwanted pregnancies and sexually transmitted infections (STI). Pre-marital sexual activity seems to be increasing among university students in Asia and Africa as a result of many factors, such as rapid urbanisation and exposure to mass media (5–8).

Nearly half of Uganda's inhabitants are below the age of 15, and 20% are between 15 and 25 (9). Poor mental health, sexual coercion, low trust in others, and increased university enrolment are associated with risky sexual behaviour among university students (10–12). Non-regular partners, unprotected sex, and cross-generational sex among university students were reported for this group in a recent study (13).

The current use of contraception among 15- to 19-year-olds in Uganda is 6.5% and 21.3% between the ages of 20 and 24 (14). A study done at six Ugandan universities showed overall condom use to be 51%, and current use of contraceptive methods other than male condoms was 9%. This study also found that 6% of all sexually active students between the ages of 15 and 19 became pregnant (13). Adolescent pregnancy is an important social concern with long-term psychosocial and economic implications for teenage mothers. These young women can be characterised as having relinquished their chance of educational attainment, placed themselves into a lower income category, and increased the risk of having to raise a child as a single parent (15, 16). In addition, infants born to adolescent mothers are exposed to a greater likelihood of foetal death, premature delivery, low birth weight, and impaired cognitive development (17, 18).

Unintended pregnancies can also lead to unsafe abortions, which account for nearly one-third of the maternal deaths among young people (19, 20). Under Uganda's strict anti-abortion law, induced abortion is rarely permitted (19). A study of Ugandan university students has shown that 7% of all sexually active women in this group have undergone an induced abortion (13).

Uganda's health sector strategic plan for 2010–2015 addresses its policy on the procurement and distribution of contraception to all males and females but specially focuses on adolescents (21). By contrast, another study conducted in Uganda found that young people face refusal or restrictions when they request contraceptives from providers (22). Nearly one-third of the providers said that they will not supply contraceptives to individuals who are younger than 18, unmarried, still in school, and those without children, although the policy guidelines of Uganda have no such requirements. Therefore, the unwillingness to provide contraceptives due to cultural or individual biases illustrate the urgency of prioritising young people's contraceptive needs. The existing gap between reproductive health, policy, and the availability of contraception restricts actual contraceptive use.

Approximately 97% of all people of reproductive age are acquainted with at least one method of contraception in Uganda (23). However, that knowledge does not translate into behaviour, for reasons which are unclear. A large body of research suggests that socio-demographic factors (24), partner type (25–27), alcohol consumption (27–32), and age at sexual debut (33, 34) influence the use of contraceptives. However, only limited research has been conducted on the determinants of contraceptive use in Uganda, especially among university students. This study seeks to address this gap by correlating the association between socio-demographic factors, alcohol consumption, and type of partners with non-use of contraception among Ugandan university students. A micro-level approach that can determine reasons for non-use of contraceptives may facilitate the design of better interventions.

## Methods

### Study design and setting

The study used a cross-sectional study design. The data were collected in April 2010 at the Mbarara University of Science and Technology (MUST), which is a public university. The study sample was drawn from the four faculties of the university: Medicine, Science, Computer Science, and Development Studies. The sample consisted of 1,954 students who participated out of a total enrolment of 2,706. They represented 72% of all the undergraduates. Since the outcome was non-use of contraception, the data analysis was based on the subset of 1,179 students who indicated they were sexually active. Of the respondents, 58.8% were male (*n*=693) and 41.2% were female (*n*=486).

### Data collection and analysis

All the students at Mbarara University were invited to participate in the survey. The questionnaires were distributed in lecture halls to undergraduate students after they were briefed about the purpose of the study and were assured that their responses would remain confidential. Students were also informed that participation was voluntary and they could discontinue it at any time. Consent forms were distributed, signed by each student, and returned anonymously to a sealed box, as were the questionnaires. The Institutional Ethics Review Committee at MUST approved the project.

The self-administered questionnaire contained 132 questions on socio-demographic factors, socio-economic status, area of growing up, role of religion, religious affiliation in which they were raised, alcohol consumption, drug use, smoking habits, sexual behaviour (including contraceptive use), social capital, and self-rated physical and mental health. The questionnaire was a follow-up study to a previous survey done at MUST in 2005 using the same questionnaire. It was based on validated instruments in other studies and resulted from group discussions held with the students in 2005 to generate the survey (10).

### Definition of variables

#### Background variables

The group was divided into two by age and coded as ≤ 22 (‘younger’) and > 22 (‘older’). The age range of the participants was between 18 and 42 years. The median age was 23 years and the mode was 22 years, which was used as the cut-off point.

Area of growing up was categorised as rural, urban and peri-urban, or small town. The variable was then dichotomised into rural or urban, the latter combining peri-urban and small town.

Educational level of head of the household was categorised as had not finished primary school, completed primary school, completed secondary school, post-secondary school, college, university education, and other. The variable was dichotomised as ≤ primary school and > primary school.

Religious affiliation during childhood was categorised as Catholic, Protestant, Moslem, Pentecostal, Seventh-Day Adventist, Orthodox, and other. The variable was then trichotomised as Catholic, Protestant, and Other since the first two were the major religions in our study sample, and the remaining denominations were too small to be analysed separately.

The role of religion in family life while growing up was dichotomised into major (‘religion played a big role or was relatively important’) and minor (‘religion was not so important or was not important at all’).

#### Relationship variables

‘Number of boyfriends/girlfriends you have had’ was dichotomised into 0–1 and ≥ 2 partners, while ‘boyfriend/girlfriend at the moment’ was categorised as ‘yes’ or ‘no’, as in the questionnaire, and referred in the study as ‘currently in a relationship’. ‘Length of the current relationship’ was dichotomised into ≤ 1 year or > 1 year.

Type of partner was a dummy variable combining ‘number of boyfriends/girlfriends you have had’ and ‘number of lifetime sexual partners’. It was then categorised as ‘irregular partners’ and ‘regular partners’ and ‘mixed partners’.

The definition of irregular partners was based on an individual reporting having had no girlfriend/boyfriend and one or more sexual partners or an individual reporting two or more sexual partners than the number of girlfriends/boyfriends.

The definition of regular partners was based on an individual reporting having had one girlfriend/boyfriend and one sexual partner, or if they had more girlfriends/boyfriends than sexual partners.

The definition of mixed partners was based on an individual reporting having had two or more sexual partners and an equal number of girlfriends/boyfriends, or one more sexual partner than girlfriends/boyfriends.

#### Sexual behaviour variables

‘Age at sexual debut’ was dichotomised into ≤ 16 or > 16 years, while ‘number of sexual partners in the past 12 months’ was determined by the response to the direct question: ‘How many sexual partners have you had in the past 12 months?’. The variable was dichotomised into 0 to 1 or ≥ 2 partners.

#### Alcohol use

Alcohol consumption in the past 12 months was assessed by responses as four or more times per week, two to three times per week, three to four times per month, once a month or less, or never. The variable was then dichotomised into ‘risk’ for the first three alternatives and ‘no risk’ for the last two.

‘Consumption of alcohol on your latest occasion of sexual intercourse’ was categorised as ‘yes’ and ‘no’ as in the questionnaire.

#### Dependent variable

Prevention of unwanted pregnancy was assessed through responses to the question: ‘Did you use any method for avoiding pregnancy, on during your latest occasion of sexual intercourse?’. There were four alternative answers: no; yes, a condom; yes, a contraceptive pill; or yes, another method. The variable was then dichotomised so that all of the ‘yes’ answers were simply considered ‘yes’.

### Statistical analysis

The analysis was done using SPSS statistical software package Version 20.0. Stratified analysis was conducted for male and female students. The prevalence of socio-demographic factors, sexual behaviour, alcohol consumption, type of partners, and contraceptive use was calculated as a percentage. The Chi-square test was used to analyse the differences between males and females for the non-use of contraceptive methods. Logistic regression analysis was done to calculate the crude odds ratio (OR) with 95% confidence interval (CI) to determine the associations between the potential determinants and non-use of contraception. Multivariable logistic regression was used to control for confounding by step-wise adjusting for age, sexual debut, and area of origin, and educational level of head of household. The OR and 95% CI were used as measures of association. Estimates of effect modification were done as ‘departure from additivity of effects on the chosen outcome scale’ proposed by Rothman (35). We performed power calculation for our study. The prevalence's of the exposure variables ranged between 20 and 50% and the prevalence of the main outcome was in the range of 15 to 20%. The power of showing effects larger than 40% in the exposed group was more than 80%.

## Results

Table 1 gives the prevalence of all the socio-demographic factors, alcohol consumption, sexual behaviour, and the outcome variable on non-use of contraception. The respondents were almost equally divided between those above 22 years or below (51.6% and 48.4%, respectively). Nearly half of the students (51.7%) in our study were from an urban background. A large proportion of the study population (71.0%) came from families where the head of the household had had more than a primary education. Religion played an important role for 62.5% of the respondents in the sample.

**Table 1:** Prevalence of socio demographic factors, sexual behaviour, alcohol consumption, and non-use of contraception among Ugandan university students (2010)

	All		Male		Female		χ^2^
				
	*n*=1,179	%	*n*=693	%	*n*=486	%	*p* *
*Age*							
Younger ≤ 2	553	48.4	290	43.1	263	56.0	0.000
Older > 22	590	51.6	383	56.9	207	44.0	
Missing	(36)		(20)		(16)		
*Area of growing up*							
Urban	607	51.7	336	48.8	271	56.0	0.015
Rural	566	48.3	353	51.2	213	44.0	
Missing	(6)		(4)		(2)		
*Educational level of head of household*							
> Primary school	820	71.0	465	68.6	355	74.4	0.035
≤ Primary school	335	29.0	213	31.4	122	25.6	
Missing	(24)		(15)		(9)		
*Religious affiliation*							
Catholic	465	39.6	275	40.0	190	39.1	
Protestant	538	45.8	311	45.2	227	46.7	
Others	171	14.6	102	14.8	69	14.2	
Missing	(5)		(5)				
*Importance of religion*							
Major	731	62.5	400	58.2	331	68.5	0.000
Minor	439	37.5	287	41.8	152	31.5	
Missing	(9)		(6)		(3)		
*Currently in a relationship*							
Yes	923	80.2	519	77.0	404	84.7	0.001
No	228	19.8	155	23.0	73	15.3	
Missing	(28)		(19)		(9)		
*Number of relationships during lifetime*							
0–1	392	35.2	192	29.4	200	43.3	0.000
≥ 2	722	64.8	460	70.6	262	56.7	
Missing	(65)		(41)		(24)		
Length of current relationship**							
≤ 1 year	479	54.2	293	58.6	186	48.4	0.003
> 1 year	405	45.8	207	41.4	198	51.6	
Missing	(39)		(19)		(20)		
*Type of partner*							
Irregular	68	7.1	52	9.3	16	4.0	
Regular	437	45.5	208	37.1	229	57.4	
Mixed	455	47.4	301	53.7	154	38.6	
Missing	(219)		(132)		(87)		
*Age at sexual debut*							
≤ 16	255	24.0	194	30.6	61	14.2	0.000
> 16	808	76.0	439	69.4	369	85.8	
Missing	(116)		(60)		(56)		
*Number of sexual partners in past 12 months*							
0–1	680	66.4	356	58.7	324	77.5	0.000
≥ 2	344	33.6	250	41.3	94	22.5	
Missing	(155)		(87)		(68)		
*Consumption of alcohol on the latest occasion of sexual intercourse*							
No	758	83.3	425	80.0	333	87.9	
Yes	152	16.7	106	20.0	46	12.1	0.002
Missing	(269)		(162)		(107)		
*Consumption of alcohol during previous 12 months*							
No risk	970	87.4	567	84.9	403	91.2	0.002
Risk	140	12.6	101	15.1	39	8.8	
Missing	(69)		(25)		(44)		
*Contraceptive use*							
Yes	869	81.4	520	83.1	349	79.0	0.094
No	199	18.6	106	16.9	93	21.0	
Missing	(111)		(67)		(44)		
*Type of contraceptive used*							
No	199	18.3	106	16.7	93	20.6	0.094
Yes, condom	693	63.7	443	69.7	250	55.3	
Yes, contraceptive pill	63	5.8	25	3.9	38	8.4	
Yes, other method	113	10.4	52	8.2	61	13.5	
Condom and contraceptive pill	15	1.4	7	1.1	8	1.8	
Condom and other methods	5	0.5	3	0.5	2	0.4	
Missing	(91)		(57)		(34)		

* *p* value in table analysed based on sex.

** Only analysed for those currently in a relationship.

More females (84.7%) were currently in a relationship than males (77.0%). There were more females in regular relationships (57.4%), as compared to males (37.1%). We found that 51.6% of the sexually active females had been in a relationship for more than a year; the corresponding figure for males was 41.4%. Sexual debut at age 16 or below was 30.6% for males and 14.2% for females. Risky alcohol consumption over the past 12 months was 15.1% for males and 8.8% for females. A larger proportion of females (21.0%) did not use contraception than males (16.9%).

Table 2 shows an analysis of the association between the socio-demographic factors, consumption of alcohol, sexual behaviour, and non-use of contraception. Females were 30% more likely not to use contraception than males (OR 1.3, 95% CI 1.0–1.8). Male students, 22 years or below, were 70% more likely not to use contraception (OR 1.7, 95% CI 1.1–2.6). Area of growing up had a significant association with non-use of contraception for males from a rural background (OR 2.2, 95% CI 1.4–3.5), whereas this factor was not of significance for females. Students currently not in a relationship were twice as likely not to use contraception: the odds for females (OR 2.6, 95% CI 1.4–4.6) were higher than males (OR 1.8, 95% CI 1.1–2.9). Male students who had sexually debuted at age 16 or younger were 80% more likely not to use contraceptives (OR 1.8, 95% CI 1.2–2.9).

**Table 2:** Association (odds ratios [OR], 95% confidence interval [CI]) between socio–demographic factors, sexual behaviour, alcohol consumption and non-use of contraception among Ugandan university students (2010)

	Non-use of contraception, *n* (%)	All, OR	Male, OR	Female, OR
*Sex*				
Male	106 (16.9)	1 (ref)		
Female	93 (21.0)	1.3 (1.0–1.8)		
				
*Age*				
Older > 22	84 (15.5)	1 (ref)	1 (ref)	1 (ref)
Younger ≤ 22	107 (21.5)	**1.5 (1.1–2.0)**	**1.7 (1.1–2.6)**	1.2 (0.7–2.0)
				
*Area of growing up*				
Urban	82 (14.9)	1 (ref)	1 (ref)	1 (ref)
Rural	116 (22.7)	**1.7 (1.2–2.3)**	**2.2 (1.4–3.5)**	1.3 (0.8–2.0)
				
*Educational level of head of household*				
>Primary school	125 (16.9)	1 (ref)	1 (ref)	1 (ref)
≤Primary School	70 (22.8)	1.5 (1.0–2.0)	1.4 (0.9–2.2)	1.7 (1.0–2.7)
				
*Religious affiliation*				
Catholic	81 (18.9)	1 (ref)	1 (ref)	1 (ref)
Protestant	85 (17.4)	0.9 (0.6–1.3)	0.9 (0.6–1.4)	0.9 (0.5–1.5)
Other	32 (21.8)	1.2 (0.8–1.9)	0.8 (0.4–1.5)	1.9 (1.0–3.7)
				
*Importance of religion*				
Major	129 (19.6)	1 (ref)	1 (ref)	1 (ref)
Minor	67 (16.6)	0.8 (0.6–1.1)	0.9 (0.6–1.3)	0.8 (0.5–1.3)
				
*Currently in a relationship*				
Yes	140 (16.3)	1 (ref)	1 (ref)	1 (ref)
No	53 (28)	**2.0 (1.4–2.9)**	**1.8 (1.1–2.9)**	**2.6 (1.4–4.6)**
				
*Number of relationships during lifetime*				
0–1	71 (20.9)	1 (ref)	1 (ref)	1 (ref)
≥2	116 (17.3)	0.8 (0.6–1.1)	0.8 (0.5–1.2)	0.9 (0.6–1.4)
				
Length of current relationship*				
≤1 year	74 (16.5)	1 (ref)	1 (ref)	1 (ref)
> 1 year	59 (15.7)	0.9 (0.7–1.4)	0.8 (0.5–1.4)	1.0 (0.6–1.8)
*Type of partner*				
Regular	72 (17.3)	1 (ref)	1 (ref)	1 (ref)
Irregular/Mixed	74 (14.8)	0.8 (0.6–1.2)	1.0 (0.6–1.8)	0.7 (0.4–1.2)
*Age at sexual debut*				
>16	117 (15.2)	1 (ref)	1 (ref)	1 (ref)
≤16	56 (23.3)	1.7 (1.2–2.4)	1.8 (1.2–2.9)	1.8 (0.9–3.3)
				
*Number of sexual partners in the past 12 months*				
0–1	113 (17.5)	1 (ref)	1 (ref)	1 (ref)
≥2	53 (16.1)	0.9 (0.6–1.3)	0.7 (0.5–1.1)	1.4 (0.8–2.6)
				
*Consumption of alcohol on latest occasion of sexual intercourse*				
				
No	126 (17.7)	1 (ref)	1 (ref)	1 (ref)
Yes	25 (18.7)	0.9 (0.6–1.5)	1.2 (0.6–2.2)	0.6 (0.3–1.2)
				
*Consumption of alcohol during previous 12 months*				
No Risk	169 (19.2)	1 (ref)	1 (ref)	1 (ref)
Risk	18 (13.6)	0.7 (0.4–1.1)	0.5 (0.3–1.0)	1.1 (0.5–2.6)

* Only analysed for those currently in a relationship.

On the basis of these findings, a multivariable logistic regression analysis was performed (Table 3). We found an association between currently not being in a relationship and non-use of contraception that continued to be statistically significant among both males and females, even after adjusting for age, sexual debut, area of growing up, and educational level of head of household. The socio-demographic determinants of age (22 or younger), early sexual debut (16 years or below), and rural background were significant for males, but not for females.

**Table 3:** Association (odds ratios [OR], 95% confidence interval [CI]) between socio-demographic factors, sexual behaviour, and non–use of contraception among Ugandan university students (2010)

	Model 1	Model 2	Model 3	Model 4
*All*				
Currently not in a relationship	**1.8 (1.2–2.8)**	**1.8 (1.2–2.7)**	**1.8 (1.9–2.7)**	**1.8 (1.2–2.7)**
≤22 years		1.3 (0.9–1.8)	**1.3 (0.9–1.8)**	**1.5 (1.1–2.1)**
Early sexual debut			**1.5 (1.0–2.2)**	**1.6 (1.1–2.3)**
Rural				**2.0 (1.3–3.0)**
Low educational level of head of household				1.3 (0.9–1.9)
				
*Males*				
Currently not in a relationship	**1.8 (1.1–2.9)**	**1.7 (1.0–2.8)**	**1.7 (1.0 – 2.8)**	**1.7 (1.0–2.8)**
≤22 years		**1.6 (1.0–2.6)**	**1.6 (1.0–2.5)**	**1.9 (1.2–3.1)**
Early sexual debut			1.6 (1.0–2.6)	**1.7 (1.1–2.7)**
Rural				**2.2 (1.3–3.8)**
Low educational level of head of household				1.2 (0.7–2.0)
				
*Females*				
Currently not in a relationship	**2.1 (1.1–4.2)**	**2.1 (1.1–4.3)**	**2.1 (1.1–4.3)**	**2.1 (1.1–4.2)**
≤22 years		1.0 (0.6–1.7)	1.0 (0.6–1.6)	1.0 (0.6–1.8)
Early sexual debut			1.6 (0.8–3.2)	1.6 (0.8–3.2)
Rural				1.3 (0.8–2.2)
Low educational level of head of household				1.4 (0.8–2.6)

To further investigate the association between the determinants that affected non-use of contraception, we analysed sexual debut as a possible effect modifier. Table 4 illustrates a synergistic effect between currently not being in a relationship and early sexual debut in their bearing on non-use of contraception.

**Table 4:** Analysis of effect modification between sexual debut and current relationship status regarding non-use of contraception in a sample of Ugandan university students (*n*=1,179), presented as adjusted odds ratios (OR) with 95% confidence intervals (CI)

	All		Male		Female	
Non-use of contraception	*n* (%)	OR (95% CI)	*n* (%)	OR (95% CI)	*n* (%)	OR (95% CI)
In a relationship/late sexual debut	719 (63.5)	1	374 (56.1)	1	345 (74.2)	1
Not in a relationship/late sexual debut	163 (14.4)	1.4 (0.9–2.2)	103 (15.4)	1.2 (0.7–2.3)	60 (12.9)	1.8 (0.9–3.5)
In a relationship/early sexual debut	191 (16.9)	1.0 (0.6–1.5)	141 (21.1)	1.1 (0.6–1.9)	50 (10.8)	0.9 (0.4–1.9)
Not in a relationship/early sexual debut	59 (5.2)	**4.6 (2.5–8.1)**	49 (7.3)	**3.9 (1.9–7.7)**	10 (2.2)	**17.5 (3.6–84.7)**
Missing	(47)		(26)		(21)	
Total	1,179		693		486	

## Discussion

Ugandan students in our sample who were currently not in a relationship were less prone to use contraception to prevent pregnancy for both males and females. However, we found significant differences with regard to gender. Younger age (22 or below), early sexual debut (age 16 years or less), and growing up in a rural environment were associated with non-use of contraception among males, but not females.

The association between currently not being in a relationship and non-use of contraception remained significant even after adjusting for potential confounders, as did all of the gender differences cited. Students who are not in a steady relationship may be more likely to engage in unplanned sexual activity. This can lead to non-use of contraception due to issues of non-availability at the time and lack of communication (36, 37). A prior study of youth also concluded that greater levels of intimacy and better partner communication before having sex increased the odds of consistent condom and contraceptive use (26).

We found no association between type of partner and non-use of contraception. Previous research on types of partners and contraceptive use has had mixed results, with some studies showing higher use of contraceptives in steady relationships (37, 38). Steady relationships may presumably allow more time to consider the use of contraceptives since it might involve more partner communication. We did not find that the length of a relationship was related to the use of contraceptives, in contrast to other studies that concluded longer relationships were associated with a greater – although inconsistent – use of contraceptives (25, 38). However, some studies document higher odds of using contraceptives in casual relationships due to mistrust and lack of commitment over avoiding unwanted pregnancies, HIV, and other STIs (27, 39).

We found that men and women who were not in a relationship had higher odds of non-use of contraception. Regarding women, this could be due to the existing socio-cultural gender norms in Uganda where men are decision makers, including contraception in which women are in a weaker position to negotiate (4). As found in previous research, cross-generational relationships between younger women and older men are common in Uganda (40). Although our questionnaire did not ask for information about the age of sexual partners, earlier studies have shown that contraceptives are used inconsistently in such situations because younger girls lack the ability to negotiate their use (25, 26).

Sexual coercion and power relations in which young women have a ‘sugar daddy’ (a considerably older male with whom they have a sexual relationship in exchange for money or material goods) are prevalent in Uganda (41). Such an unequal relationship plays a role in decision making about contraceptives and must be addressed in any planned intervention. Also, a study conducted in 2005 at the same university showed that 29.0% of the male students and 33.1% of the female students reported having had some experience of sexual coercion, underscoring the potential role of this factor in the non-use of contraceptives (11).

Our findings imply that even educated women may not have the power to negotiate contraceptive use (42). A study done on Ugandan adolescents found that there could be socio-cultural barriers that may stigmatise obtaining contraceptives. Cost may also play a role in limiting use, especially among unmarried university students. More females than males reported fear or embarrassment for purchasing contraceptives (43, 44).

Socio-demographic determinants like age (22 years or younger) and rural background were found to have an association with non-use of contraception among males. This may be due to the risk-taking behaviour of young boys or the lack of access to contraceptives at the time of unplanned sexual activity (4). It is also possible that students who have moved from a morally restrictive rural environment to a more liberal urban one are introduced to university parties and alcohol that further expose them to risky sexual activities (45).

We were surprised to find that alcohol consumption did not have an association with non-use of contraception in our sample. Several studies had concluded that alcohol abuse by university students was associated with elevated rates of risky sexual behaviour with regard to inconsistent condom use (28–31, 46). Alcohol consumption has been said to have a negative impact on the use of contraception in casual relationships due to its potential for disrupting efficient communication (31). In such relationships both instigatory cues (arousal) and inhibitory cues (restraint) are presumed to be high, as each partner weighs potential sexual health risks. According to the alcohol myopia theory, alcohol may limit a person's capacity to weigh negative outcomes (31, 47, 48). Thus, alcohol use may lead to greater risk-taking behaviour, including that of incurring an unwanted pregnancy.

Early sexual debut was found to have an independent association with non-use of contraception for males. This agrees with research showing that those who experience sex at an early age are less likely to use contraception than those who initiate sexual activity later in life (33, 34, 49, 50). Many sexually active young people are not prepared to protect themselves from pregnancy and do not use contraception because they do not know where to obtain them, lack of knowledge of HIV and other STIs, or because of perceived barriers to accessing healthcare services (44). Age of sexual debut is also a strong determinant of a person's future sexual lifestyle, as shown in a study of Chinese university students in which early sexual debut was associated with increased risk of STIs, unwanted pregnancies, induced abortions, multiple partners, and reduced condom and oral contraceptive use (7). We also found synergy between sexual debut at 16 years or below and not being in a relationship in regard to non-use of contraception.

### Strengths and limitations

The study design was cross-sectional, which led to the causal direction being open regarding some of the associations. The statistical power of the study was adequate for the main analysis but low for the analysis of synergy, and no formal test of statistical significance was made for those analyses. The information students provided on their sexual activity and contraceptive use was retrospective, which could have led to recall bias. Since the focus of this survey was not to determine prevalence or risk factors for contraceptive use, data were not collected on types of contraception, use on occasion of first sexual intercourse, or lifetime use. We sought to control for the possible confounding effect of socio-demographic factors and other known determinants of risky sexual behaviour in the multivariable analysis. Dichotomising the variables might have led to data loss. Additional study may further qualify how dynamics of relationships affect contraceptive use among university students and lead to more targeted interventions to promote sexual health.

## Conclusion

Non-use of contraception among Ugandan university students differs for men and women. The diversity of socio-demographic factors regarding risky behaviours seems to be pertinent to males but not females. The most plausible explanation for the observed differences could be the existing gender power relations in Ugandan society. Decision-making power for contraceptive use largely appears to rest with males, especially among those who were not in a steady relationship. Therefore, sexual and reproductive health programmes and policies should focus on existing gender imbalances. This might be accomplished by developing strategies to promote participation among young men and women. This could be done through imparting women with communicating skills to negotiate contraceptive use, and by creating awareness on gender issues, particularly among men.
